# Ms2lda.org: web-based topic modelling for substructure discovery in mass spectrometry

**DOI:** 10.1093/bioinformatics/btx582

**Published:** 2017-09-14

**Authors:** Joe Wandy, Yunfeng Zhu, Justin J J van der Hooft, Rónán Daly, Michael P Barrett, Simon Rogers

**Affiliations:** 1 Glasgow Polyomics University of Glasgow, Glasgow, UK; 2 Wellcome Centre for Molecular Parasitology, Glasgow, UK; 3 School of Computing Science, University of Glasgow, UK

## Abstract

**Motivation:**

We recently published MS2LDA, a method for the decomposition of sets of molecular fragment data derived from large metabolomics experiments. To make the method more widely available to the community, here we present ms2lda.org, a web application that allows users to upload their data, run MS2LDA analyses and explore the results through interactive visualizations.

**Results:**

Ms2lda.org takes tandem mass spectrometry data in many standard formats and allows the user to infer the sets of fragment and neutral loss features that co-occur together (Mass2Motifs). As an alternative workflow, the user can also decompose a data set onto predefined Mass2Motifs. This is accomplished through the web interface or programmatically from our web service.

**Availability and implementation:**

The website can be found at http://ms2lda.org, while the source code is available at https://github.com/sdrogers/ms2ldaviz under the MIT license.

**Supplementary information:**

Supplementary data are available at *Bioinformatics* online.

## 1 Introduction

A typical tandem mass spectrometry analysis (MS/MS) can easily produce fragmentation spectra for thousands of molecules. Analysis of this data is challenging, and traditionally the use of fragmentation data has been limited to performing database searches for identification. Recently, we developed MS2LDA (van der Hooft *et al.*, 2016), a method inspired by text modelling, to decompose fragmentation spectra into sets of conserved fragment and neutral loss features (called *Mass2Motifs*). As text documents can be decomposed into sets of co-occurring words (topics) MS2LDA decomposes each molecule into sets of Mass2Motifs, potentially indicative of structural families. The code provided with the original publication allowed MS2LDA to be used but the resulting analysis process was quite labour intensive and did not allow for data to be shared across experiments. Here, we present ms2lda.org: a Web application for MS2LDA analysis in which users can upload MS/MS data, use MS2LDA to extract Mass2Motifs, annotate and store annotations for the extracted Mass2Motifs (either by manual inspection or comparison with other experiments) and interactively exploring the decomposition results (including linking MS1 intensity changes with Mass2Motifs). The resulting software makes MS2LDA accessible to a much wider community of researchers and will ultimately result in an increasing database of Mass2Motifs that can be used to structurally characterize more and more unknown molecules.

## 2 Methods and implementation

The system is implemented in Python with a minimal set of dependencies. Django is used as the Web application framework, whereas a job queue (Celery) is used to execute analyses asynchronously. As in the original publication, variational inference to discover Mass2Motifs is implemented in Numpy/SciPy, and interactive visualizations are performed in JavaScript (D3.js). Features are extracted from mzML fragmentation files using the pymzML library and stored in a relational database (PostgreSQL) alongside inferred Mass2Motifs and associated metadata.

Two analysis workflows are implemented in our system—the original MS2LDA and a new feature, MS2LDA decomposition. In the former, fragment and neutral loss features are extracted from the uploaded fragmentation file [mzML (alongside an optional peaklist file to filter the extracted precursors, see Supplementary Section S1), MSP, or MGF formats] and stored inside the database. MS2LDA is performed on the loaded spectra, and the results are stored in the database for visualization. Various filtering options are provided to allow for filtering of the loaded spectra, and the user can also specify algorithm properties (number of Mass2Motifs and number of algorithm iterations). To let users control their data, we require users to register before uploading data. However, we have created a guest user account to allow potential users to explore the system prior to registration.

In the second workflow (decomposition), users can decompose a data set (uploaded in one of the formats mentioned above) onto a previously defined set of Mass2Motifs. This can be done through the ms2lda.org web interface, or programmatically through a Web service (see Supplementary Section S2). This previously unpublished feature allows users to rapidly decompose molecular spectra based on the presence of Mass2Motifs from our database and, where these Mass2Motifs are characterized, annotate the molecules.

**Fig. 1:**
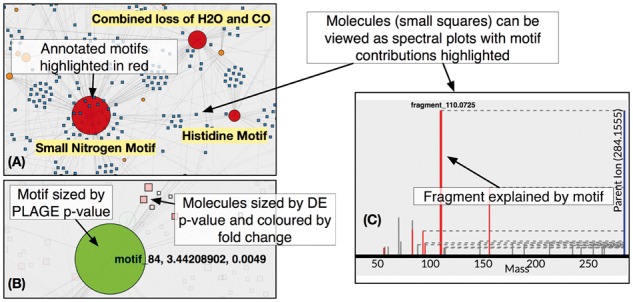
An example of ms2lda.org visualization. The starting point for visualization is the network graph (**A**), nodes represent Mass2Motifs (circle) and molecules (squares). Mass2Motif nodes are sized according to the number of connected molecules. Edges represent connections between molecules and Mass2Motifs (above a certain threshold). When MS1 analysis is available (**B**), Mass2Motif nodes can be coloured according to their PLAGE scores and sized according to the PLAGE *P*-values. Similarly, molecules can be coloured according to fold change and sized according to their differential expression *P*-value. Selecting a Mass2Motif node in the graph reveals the spectral plot for its associated molecules (one example for the histidine Mass2Motif is shown in **C**). Fragments and losses that are explained by the selected Mass2Motif are highlighted in red (Color version of this figure is available at *Bioinformatics* online.)

In both workflows, analysis results can be shared to multiple users (in read-only or edit mode), allowing for joint analysis of a data set. For each analysis, the summary page lists inferred Mass2Motifs and molecules explained by these motifs. Additionally, we also provide an interactive visualization feature including a network view (see Fig. 1), spectral plots (with Mass2Motif contributions highlighted) and feature statistics (e.g. proportion of a particular fragment‘s total intensity explained by a particular Mass2Motif).

Additional new functionality is the ability to store and edit Mass2Motif annotations, which appear in all visualizations. In addition, where MS1 intensity information is available for the fragmented molecules (across multiple samples), case versus control analyses can be performed. We have implemented the PLAGE method (Tomfohr *et al.*, 2005) to allow variation in MS1 intensity to be used to assess if Mass2Motifs themselves are differentially expressed. Results from such analyses can be overlaid on the network graph (Fig. 1).

Matching Mass2Motifs discovered in one analysis with Mass2Motifs found and potentially annotated from previous analyses avoids re-annotation. As a new feature, we offer matching functionality which allows users to compare the Mass2Motifs found in their data with those in our growing database of (often annotated) Mass2Motifs. Users can view candidate matches and link Mass2Motifs where they agree with the match. When linked, annotations are transferred between matched pairs. Ms2lda.org also includes a growing library of pre-annotated Mass2Motifs from the Massbank and GNPS data sets (van der Hooft *et al.*, 2016) for matching and decomposition.

## 3 Conclusion

Ms2lda.org allows users to decompose and annotate MS/MS data with MS2LDA (van der Hooft *et al.*, 2016). In addition to the original MS2LDA functionality, we provide a new visualization screen, the ability to store annotations, decomposition, motif matching and MS1 analysis. We believe that ms2lda.org provides an easy entry to perform topic modelling on MS/MS based metabolomics data and visualize and analyse the resulting model. We are working on various extensions to the system, which will be incorporated as they are available. These include a method for exporting Mass2Motifs into a MassBank-compatible format and automated Mass2Motif characterization.

## Funding

This work was supported by the Wellcome Trust [Grant No. 105614/Z/14/Z to J.J.J.vdH. and R.D.]; Wellcome Trust core grant to the Wellcome Trust Centre for Molecular Parasitology [104111/Z/14/Z to M.P.B.]; and BBSRC [Grant No. BB/L018616/1 to S.R.].

*Conflict of interest*: none declared.

## Supplementary Material

Supplementary Data
