# Supplementary material for: Ms2lda.org: web-based topic modelling for substructure discovery in mass spectrometry
Source: Bioinformatics. 2017 Sep 14;34(2):317–8. doi: 10.1093/bioinformatics/btx582 (PMC5860206; doi:10.1093/bioinformatics/btx582)
Supplement: Supplementary Data [file btx582_supporting_info.pdf]

Ms2lda.org: web-based topic modelling for substructure discovery in mass spectrometry

Joe Wandy[a], Yunfeng Zhu[b], Justin J.J. van der Hooft[a], Rónán Daly[a], Michael P. Barrett[a,b], and Simon Rogers[c,*]

[a]Glasgow Polyomics, University of Glasgow, Glasgow, United Kingdom
[b]Wellcome Centre for Molecular Parasitology, Glasgow, United Kingdom
[c]School of Computing Science, University of Glasgow, Glasgow, United Kingdom

September 8, 2017

1 Supplementary Section S1: MS1 Peak-list Format

The required file format for the MS1 peak list is as follows:

A .CSV file with header line:

`("..., mass, RT, samplename_1, samplename_2,..."), delimiter: '.`

The mass column must have the column header of either '*mass*' or '*mz*' (case-insensitive). Retention time is assumed to be the column immediately after mass, and should be in seconds. Any columns before mass (e.g. containing peak IDs from other software) will be ignored. All columns after retention time will be interpreted as MS1 intensities, with one column per sample. In the .CSV file, missing data can be stored as empty or NA, for examples, any of the following is valid:

- 123.45„567.89
- 123.45,NA,567.89

Any intensities that are negative values, cannot be interpreted as a float, or are empty will be ignored and not be stored for further analysis.

2 Supplementary Section S2: Decomposition Application Programming Interface (API)

The batch decomposition API is documented here: http://ms2lda.org/api. It allows a user to send a bunch of spectra to the server to be decomposed onto a particular motifset. The spectra are passed as the arguments to a POST request to the following URL:

```
http://ms2lda.org/decomposition/api/batch_decompose/
```

The argument should be a dictionary with the following two '<*key, value*>' pairs:

- Key: '*motifset*', value: name of the motifset to decompose onto, e.g. '*massbank_ motifset*'
- Key: '*spectra*', value: the spectral information, pickled into a string (using e.g. json.dumps)

The spectra value should be a list, with one item per spectra. The item should be a tuple with three elements:

```
(string: doc_name, float: parentmass, list: peaks)
```

Peaks is a list of tuples, each representing a peak in the form

```
(float: mz, float: intensity)
```

2.1 Python Example

For example in Python, using the requests package:

```
import requests
import json

spectrum = ('spec_name',188.0818,[(53.0384,331117.7),
(57.0447,798106.4),
(65.0386,633125.7),
(77.0385,5916789.799999999),
(81.0334,27067.0),
(85.0396,740633.6)])

spectra = [spectrum] # or add more to the list

args = {'spectra': json.dumps(spectra), 'motifset': 'massbank_motifset'}

url = 'http://ms2lda.org/decomposition/api/batch_decompose/'

r = requests.post(url,args)
```

Because this is computationally intensive, the decomposition is run as a scheduled task. Therefore the POST request doesn't return the results immediately. Instead it returns some summary, including the ID of the results entry. To get the results (in JSON), do the following:

```
result_id = r.json()['result_id']
```

```
url2 = ‘http://ms2lda.org/decomposition/api/batch_results/{}/’.format(result_id)
r2 = requests.get(url2)
print r2.json()
```

If ’r2.json()’ has a ’status’ field, it means the job is still running or waiting. If not, you get a dictionary back with the document names as keys and a list as the value. Each list element has the form:

```
‘(string:globalmotifname, string:originalmotifname, float:theta, float:overlap_score)’
```

3 Supplementary Section S3: System Performance

At the moment, Ms2lda.org is hosted on a virtual private server (DigitalOcean) having a 2.4 GHz dual-core processor and 2 GB of RAM (note that these hardware specifications can be increased easily based on future requirements). The queuing system (Celery) is configured to execute one job at a time on one CPU core, leaving the other core free to service Web requests responsively.

The complexity of mean-field variational inference for LDA is $O(NKV)$, where N is the number of fragmentation spectra, K the number of Mass2Motifs and V the number of unique fragment/loss features. These are the three main factors affecting the running time of an analysis. As an example of actual running time, the LDA analysis of an mzML file containing 1453 fragmentation spectra and 7397 fragment/loss features finishes in approximately an hour. This includes all steps of the pipeline, such as feature extraction, Mass2Motif inference, and the loading of results into the database for visualisation.
